# Characteristics of within-household varicella transmission events associated with school outbreaks in Shanghai, China, 2009–2018

**DOI:** 10.1017/S0950268820000448

**Published:** 2020-02-14

**Authors:** Yuan-Fang Chen, Qi Zhou, Jing-Yi Liu, Rui-Jie Gong, Shu-Qian Mao, Zhuo-Jun Ye, Qiang-Song Wu

**Affiliations:** 1 Xuhui District Center for Disease Control and Prevention, Shanghai 200237, China; 2 School of Public Health, Shanghai University of Traditional Chinese Medicine, Shanghai 201203, China

**Keywords:** Household, outbreak, post-exposure prophylaxis, varicella vaccine, varicella

## Abstract

Transmission of varicella occurs frequently in schools and households. We investigated the characteristics of varicella cases derived from within-household transmission and the modes of varicella transmission between school and household settings in Shanghai, China, from 2009 to 2018. Within-household transmission occurred in 278 households, of which 134 transmission events were between children. Sixty-one household varicella transmission events may be attributed to isolation procedures for infected students during school outbreaks, and 7.6% of school outbreaks were caused by schoolchildren cases derived from within-household transmission. The frequency of ‘school-household-school’ transmission adds an additional layer of complexity to the control of school varicella outbreaks. Administration of varicella vaccine as post-exposure prophylaxis after exposure is considered to be an effective measure to control varicella spread within households and schools.

Varicella is an infectious disease caused by varicella-zoster virus. Transmission of varicella occurs frequently in both households and schools. Most varicella outbreaks occur in schools, and due to the outbreak control measure (isolation of cases) and the high secondary attack rate in households (70–87%) [1, 2], infected students may trigger within-household transmission. Secondary transmission of varicella on a school bus was reported arising from an infectious and unvaccinated student who contracted varicella via within-household transmission [3]. Therefore, ‘school-household-school’ transmission may produce additional school outbreaks and make outbreaks harder to control. However, to our knowledge, no studies have been reported on within-household transmission of varicella in China, resulting in a lack of data to support the government in formulating outbreak prevention and control strategies.

Shanghai is a metropolitan city in the east of China. A voluntary single-dose varicella vaccine (VarV) schedule has been recommended in Shanghai since 1998 for children aged ≥12 months, and routine two-dose vaccination was included in the immunisation programme in August 2018 for children aged 1–4 years [4]. Although single-dose VarV coverage among schoolchildren was 88%, school outbreaks continued to occur frequently in Shanghai from 2006 to 2017 [4]. This study aimed to investigate the characteristics of varicella cases in households and the modes of varicella transmission between school and household settings in Shanghai. Our overall goal was to provide data to support varicella outbreak control.

Since 2006, all clinicians are required to register varicella case information, including the guardian's name, contact number and address, in the National Infectious Disease Reporting System (NIDRS) within 24 h of a varicella diagnosis in Shanghai, China. Clinical varicella case definitions (development of an acute generalised maculopapulovesicular rash following exposure to varicella) are widely used in China for rapid identification of cases. The average incubation period of varicella is 14 days (range 10–21 days). Therefore, an infected individual was considered to be the index case when no previous varicella cases had occurred in the setting within the maximum incubation period (21 days). Cases occurring after the index case and with an interval of longer than the minimum interval (10 days) were considered secondary cases. School-based varicella outbreaks were defined as two or more epidemiologically-linked clinical cases of varicella reported in a school within 21 days. Within-household transmission between cases was defined using the following three criteria: (1) cases were reported in NIDRS from 2009 to 2018, (2) cases resided in the same household, as assessed by any two repeated records in the guardian's name, contact number or contact address, and (3) two or more cases occurred in the same household within 21 days. We divided cases into four groups: non-schoolchildren (age 0–2 years), schoolchildren (age 3–18 years) and adults (age >18 years). Individuals 0–18 years of age were considered children.

Cases of within-household transmission were matched with the database of school outbreak-related varicella cases to determine whether they were associated with a school outbreak, and further obtained their vaccination history from the Shanghai Immunization Information System. A flow diagram of the study is shown in Figure 1. Data were analysed using SPSS 18.0, and differences between groups were assessed using *χ*^2^ tests or Student's *t* tests, as appropriate.

**Fig. 1.**
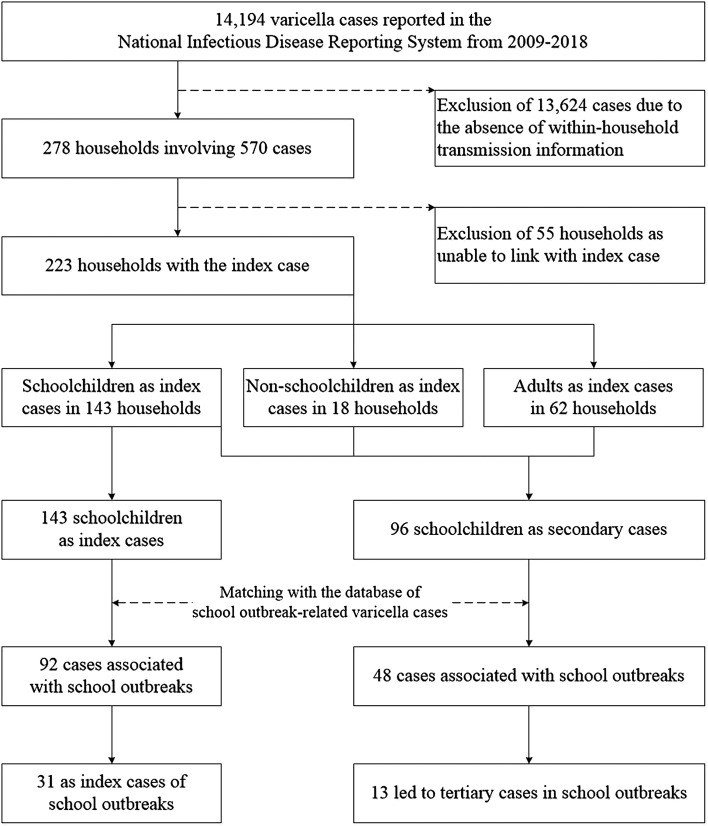
Inclusion criteria for within-household varicella transmission events and linkage of cases with school outbreaks in Shanghai, China, from 2009 to 2018.

A total of 14 194 clinically confirmed varicella cases were reported in Shanghai from 2009 to 2018. There were 278 households containing two or more cases within 21 days (Fig. 1). The majority of households (95.0%) reported only two cases of varicella, while the remaining households (5.0%) reported more than two cases. In 134 households, the cases were all children, while 116 households contained both child and adult cases and 28 households contained only adult cases (Table 1).

**Table 1.** Characteristics of within-household varicella transmission events in Shanghai, China, from 2009 to 2018

Characteristics	Households (%) *n* = 278
No. of cases in a single household	
2 cases	264 (95.0)
>2 cases	14 (5.0)
Case composition	
Children	134 (48.2)
Children and adult^a^	116 (41.7)
Adult	28 (10.1)
Interval between the first two cases	
<10 days	55 (19.8)
⩾10 days	223 (80.2)
Age group of index case^b^	
Non-schoolchildren	18 (8.1)
Schoolchildren	143 (64.1)
Adult	62 (27.8)
Outbreak-related household	
Yes	151 (54.3)
No	127 (45.7)

a Eight households involved two children and one adult.

b Fifty-five households were excluded because the index case could not be determined.

In total, 570 cases were reported in the 278 households, accounting for 4.0% of the total cases in Shanghai from 2009 to 2018. The mean age of these 570 cases was 14.43 ± 12.13 years and 275 (48.2%) were male. More than half of cases were schoolchildren (53.9%; mean age 8.73 years), followed by adults (31.4%; mean age 30.43 years) and non-schoolchildren (14.7%; mean age 1.20 years).

The mean interval between the first two cases in a single household was 12.53 ± 5.85 days, and the interval in the majority of 223 (223 households, 80.2%) was longer than 10 days (Table 1). Of the 223 households containing index cases, 107 involved child-to-child transmission. The mean age of index cases was 8.39 years in these 107 households, and the mean age of the 110 secondary cases was 6.33 years (*t* = 3.532, *P* = 0.001). A total of 227 child cases were reported in these 107 households, and 116 cases had a documented history of varicella immunisation. There was no difference in vaccination coverage between index and secondary cases (*χ*^2^ = 0.333, *P* = 0.564).

There were 576 varicella outbreaks in schools in Shanghai from 2009 to 2018, and 151 (54.3%) household varicella transmission events were associated with school outbreaks (Table 1). Within the 223 households containing index cases, nearly two-thirds of index cases were schoolchildren (64.1%), followed by adults (27.8%) and non-schoolchildren (8.1%) (Table 1). A total of 239 cases among schoolchildren were reported in these 223 households, including 143 index cases and 96 secondary cases (Fig. 1). Of the 143 schoolchildren index cases, 92 (64.3%) were involved in school outbreaks. Among 92 school-related cases, the 31 index cases of within-household transmission were also the index cases for school outbreaks (Fig. 1). Sixty-one (27.4%) household varicella transmission events were caused by schoolchildren infected during school outbreaks. Of the 96 schoolchildren secondary cases, 48 (50.0%) were associated with school outbreaks and 13 (27.1%) were the index cases for school outbreaks (Fig. 1). In within-household transmission events, the proportion of school outbreak-involved cases among index cases was higher than that among secondary cases (64.3% *vs.* 50%; *χ*^2^ = 4.865, *P* = 0.027). A total of 44 (7.6%) school outbreaks were caused by schoolchildren cases derived from within-household transmission (Fig. 1).

In this study, we described the characteristics of cases derived from within-household varicella transmission and the association of varicella transmission between household and school settings in Shanghai, China, from 2009 to 2018. Among the 278 households studied, about half of within-household transmission events occurred between children and about 40% of within-household transmission events occurred between children and adults. The lower rate of transmission between children is likely due to the high proportion of single-child households (86.0–94.2%) [4]. However, schoolchildren cases tend to have higher visitation rates than adults [5], which may contribute to the underestimation of within-household transmission between children and adults. Consistent with some other studies [1, 6], we found that index cases were significantly older than secondary cases during within-household transmission between children. This may be explained by an increased risk of infection for the first-born child enrolled in schools and with lower VarV coverage [1, 7]. In addition, our study confirmed that nearly 30% of household varicella transmission events were caused by cases infected during school outbreaks, and that 7.6% of school outbreaks were initiated from household-transmitted varicella. With the implementation of China's two-child policy, the proportion of schoolchildren with siblings is increasing [4], which may increase the risk of varicella transmission between schools and households. Therefore, ‘school-household-school’ transmission makes school outbreak control more difficult.

Case isolation is still the most common varicella control measure during school outbreaks. Results from some studies suggested that secondary cases in households were more severe than the index case, experiencing a higher number of pox, higher fever and more frequent pneumonia [6, 8]. Cases among isolated students may lead to within-household transmission during school outbreaks, especially when the isolated students have siblings. It was reported that children with one-dose of VarV had lower secondary attack rates than unvaccinated children during household transmission (15% *vs.* 72%) [9]. Administration of VarV as post-exposure prophylaxis (PEP) has proved to be highly effective for the prevention of secondary varicella cases in schools, households and shelters [10–12]. Early studies have shown that the vaccine effectiveness of VarV administered as PEP within 5 days of exposure (to prevent varicella disease of any severity) was extremely variable (9–100%) [13–15] but was highly effective for the prevention of severe disease (88–100%) [13, 14]. Therefore, improving VarV coverage among family members prior to exposure or administration of VarV as PEP after exposure could reduce the secondary attack rate during household transmission.

There were some limitations to our study. First, cases involving household varicella transmission events in this study only represent 4.0% of the cases. Second, this was a retrospective study based on data from NIDRS, which may result in an underestimate of the number of within-household transmission events due to changes in guardian information and the low visitation rates of adult cases. Third, our results may not be generalisable to other areas due to differences in population composition as well as in vaccination policies.

In conclusion, nearly 30% of household varicella transmission events may be due to measures to isolate infected students during school outbreaks. Secondary cases derived from within-household transmission can also lead to tertiary cases in schools. Therefore, the frequent occurrence of ‘school-household-school’ transmission among schoolchildren makes school outbreak control more difficult. Governments must pay careful attention to within-household spread of varicella during quarantine of schoolchildren. Vaccination is considered to be an effective measure to control the secondary attack rate during household transmission, and administration of VarV as PEP within 5 days after exposure is another measure to control varicella spread within households. In the future, studies are necessary to determine whether the newly implemented two-dose VarV schedule is effective in reducing the frequency of ‘school-household-school’ transmission among schoolchildren.
